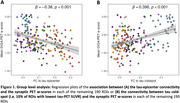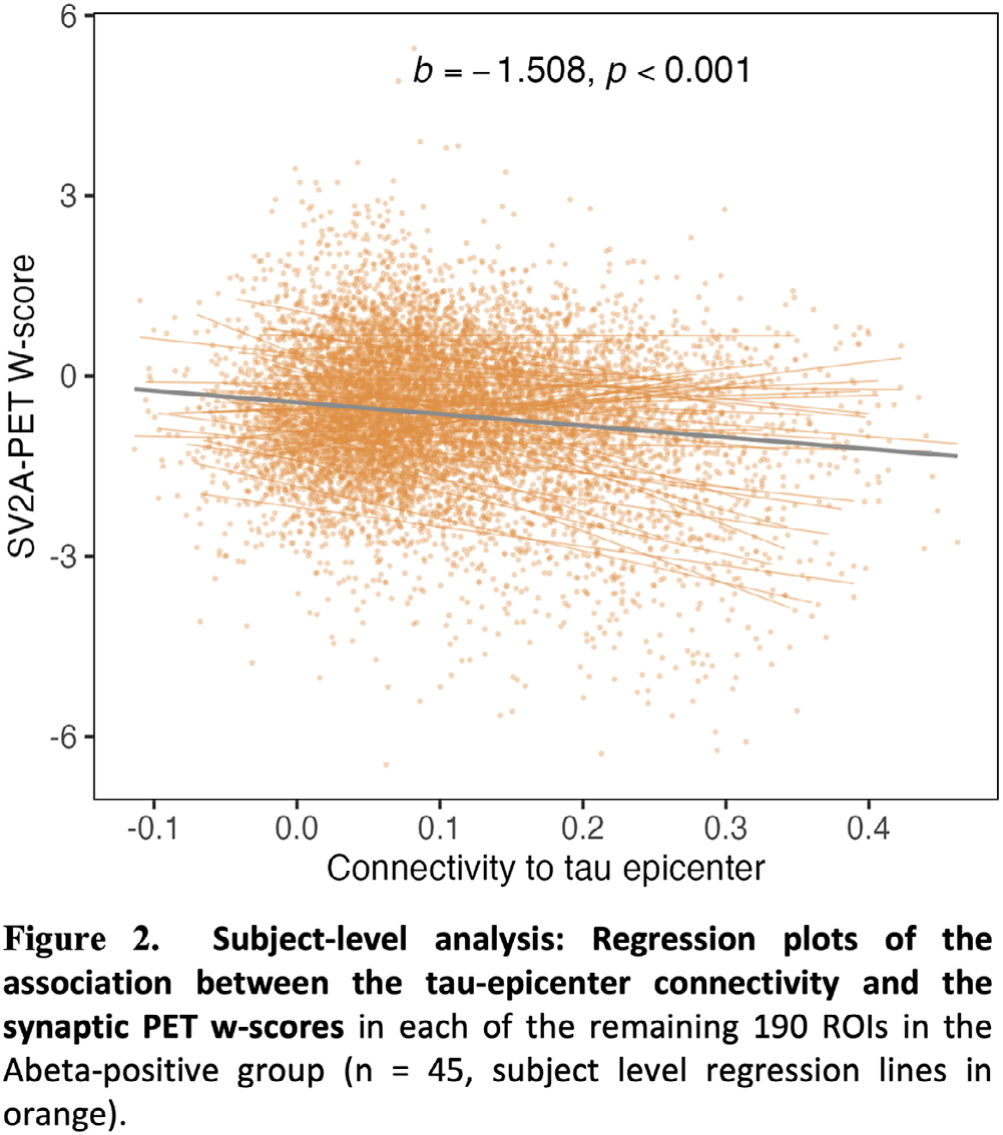# Connectivity to epicenters of tau accumulation contributes to regional synaptic loss in Alzheimer’s disease

**DOI:** 10.1002/alz.090863

**Published:** 2025-01-09

**Authors:** Ying Luan, Weiyi Wang, Yihui Guan, Nicolai Franzmeier, Fang Xie, Michael Ewers

**Affiliations:** ^1^ Zhongda Hospital, School of Medicine, Southeast University, Nanjing China; ^2^ Huashan Hospital, Fudan University, Shanghai China; ^3^ Huashan Hospital, Fudan University, Shanghai, Shanghai China; ^4^ Munich Cluster for Systems Neurology (SyNergy), Munich, Bavaria Germany; ^5^ University of Gothenburg, The Sahlgrenska Academy, Institute of Neuroscience and Physiology, Psychiatry and Neurochemistry, Gothenburg Sweden; ^6^ Institute for Stroke and Dementia Research, Ludwig‐Maximilians‐Universität München, LMU München, Munich Germany; ^7^ German Center for Neurodegenerative Diseases (DZNE), Munich, Bavaria Germany; ^8^ Institute for Stroke and Dementia Research, University Hospital, Ludwig‐Maximilian‐University, Munich Germany

## Abstract

**Background:**

Alzheimer’s disease (AD) is associated with substantial synaptic loss potentially due to synaptotoxicity of fibrillar tau, but the association between tau deposition and synaptic loss remains unclear. Based on previous observations that pathology spreads preferentially between closely connected regions, we tested in the current multi‐PET tracer study the hypothesis that synaptic loss propagates to regions closely connected to epicenters of high tau accumulation.

**Method:**

We assessed 18F‐SynVesT‐1 PET as a measure of synaptic vesicle glycoprotein 2A (SV2A), and 18F‐flortaucipir tau‐PET in fourty‐five 18F‐florbetapir‐PET‐positive (Aβ+) subjects with MCI or AD dementia, and 23 cognitivly normal (CN) Aβ‐ controls. All PET scans were spatially normalized and parcellated into 200 ROIs. Adopting our previously established connectivity‐based prediction model (Franzmeier et al. Nat Commun 2020), we computed tau‐epicenter connectivity maps by projecting regions of highest tau‐PET SUVR (top 10% of group‐average tau‐PET ROIs) onto a normative resting‐state fMRI derived connectivity template from the Human Connectome Project. The tau‐epicenter connectivity map was projected onto the group‐average synaptic PET ROI w‐scores (standardized SUVR difference between Aβ+ vs Aβ‐ group) to test in linear regression analysis, epicenter connectivity as a predictor of synaptic PET w‐scores in connected ROIs. As a control, we conducted an additional regression analysis, using this time connectivity to cold spots of tau‐accumulation (10% of ROI with lowest tau‐PET uptake) instead of epicenter‐connectivity as a predictor. We repeated the analyses at the subject level, controlling for the effect of age, gender, clinical diagnosis and global amyloid‐PET uptake.

**Result:**

Higher functional connectivity to the tau‐epicenter was associated with lower synaptic PET binding in the connected ROIs (β = ‐0.380, p < 0.001, Figure 1A). In contrast, functional connectivity to the tau coldspot ROIs was associated with more preserved synaptic PET binding in the connected ROIs (β = 0.396, p < 0.001, Figure 1B). Subject‐level analysis showed consistent findings (Figure 2 for tau‐epicenters).

**Conclusion:**

Synaptic loss in AD occurs in a connectivity‐dependent manner, where closer functional connectivity to regions of high tau is associated with stronger loss in synaptic PET. Putative underlying mechanisms are the higher tau in tau‐epicenter connected regions or functional decline in the connected regions.